# Intracellular drug binding affinities by NMR

**DOI:** 10.1107/S2059798321010135

**Published:** 2021-10-01

**Authors:** Tobias Madl

**Affiliations:** a Gottfried Schatz Research Center for Cell Signaling, Metabolism and Aging, Molecular Biology and Biochemistry, Medical University of Graz, Neue Stiftingtalstrasse 6/VI, 8010 Graz, Austria

**Keywords:** in-cell NMR, ligand binding affinity, compound–target interactions, drug development

## Abstract

A commentary on the article by Luchinat *et al.* [(2021), *Acta Cryst.* D**77**, 1247–1258] where they describe an approach to identify the details of a compound binding to a molecular target using in-cell NMR to provide intracellular binding affinities.

Most small-molecule drugs fulfill their function in cells, but studying direct compound–target interactions in a cellular environment is challenging. Nevertheless, this is crucial information guiding drug development where drug engagement at the desired site of action and potential off-target binding and activity need to be assessed as early as possible. Rather than intracellular binding affinities, available approaches rely on activity-based cellular assays, and provide a downstream and indirect impact of the respective compounds on target activity. In 2006, several seminal publications proposed a first solution towards this problem by demonstrating that nuclear magnetic resonance (NMR) spectroscopy can be used to study proteins in living cells (Selenko *et al.*, 2006 ▸; Serber *et al.*, 2006 ▸). Since then, ‘in-cell NMR’ has been successfully applied to study proteins, their (post-translational) modifications and interactions (reviewed in Luchinat & Banci, 2017 ▸; Selenko & Wagner, 2007 ▸; Pastore & Temussi, 2017 ▸; Siegal & Selenko, 2019 ▸). More recently, several groups have shown that in-cell NMR can be used to study interactions of proteins with small-molecule compounds in living cells (DeMott *et al.*, 2018 ▸; Luchinat *et al.*, 2020 ▸). However, whether intracellular compound-target interactions can be determined quanti­tatively in terms of binding affinities has remained unknown.

In this issue of *Acta Cryst. D*, Luchinat *et al.* report that NMR spectroscopy can provide intracellular binding affinities in the nanomolar range provided that a reference compound with a known dissociation constant (*K*_d_) is available (Luchinat *et al.*, 2021 ▸). Using the well studied human carbonic anhydrase II as a model system, Luchinat *et al.* assessed the binding and competition of a set of four small-molecule inhibitors with known binding affinities *in vitro* and in living cells. To this end, the authors expressed [^15^N]-His-labeled carbonic anhydrase II in HEK293 T cells and followed changes in chemical shifts of ^1^H,^15^N cross-peaks by NMR spectroscopy (Fig. 1 ▸). This has the advantage that NMR signals specific for carbonic anhydrase II can be observed even in a complex cellular background. The authors carried out competition experiments either from a series of independent cell samples or from a single real-time NMR bioreactor run. Excitingly, Luchinat *et al.* found that the intracellular affinities were similar to those obtained *in vitro*.

Whereas it still remains to be shown whether this approach can be applied to other target proteins, these findings promise that in-cell NMR can provide a new route to the determination of intracellular ligand-binding affinities for other target proteins in the future. In principle, this approach should be applicable to all target proteins provided that (i) they can be expressed or introduced in cells isotopically labeled at sufficient concentrations (>10 µ*M*); (ii) there is a lack of binding to other cellular components which would cause extensive broadening of NMR signals; (iii) there is sufficient protein stability; (iv) there are well separated NMR signals in the cellular background; (v) sufficient chemical shift differences are induced by ligand-binding and (iv) there is strong ligand binding. Moreover, the NMR-based approach is not limited to proteins, but could be extended to other biomolecules such as nucleotides and other isotope labeling schemes.

The real-time NMR bioreactor setup described promises to be well suited to obtain comprehensive and time-resolved datasets for cellular compound uptake and, at the same time, intracellular compound–target interactions. Remarkably, samples of HEK293 T cells could be kept viable and metabolically active for up to 60 h as shown in this study. An additional and great advantage of this setup is that concentrations of reference and test ligands can be changed easily, and the response of the intracellular compound concentrations and target binding can be followed in real-time inside the NMR spectrometer.

Identifying the intracellular details of a compound binding to a molecular target promises to become a solvable problem with the approach described by Luchinat *et al.*, provided the aforementioned requirements are met. In future studies it will be very interesting to see if in-cell NMR can be used for compound screening, for example by testing ligands or ligand mixtures with unknown cellular uptake or target affinity. Off-target compound binding is a common problem faced during drug development, and might be tractable by in-cell NMR. It remains to be investigated what the requirements for detectable off-target binding are, for example in terms of binding affinities and expression levels of off-targets. Given the flexibility of NMR spectroscopy, future studies might even go beyond compound-centered disease-related studies and enable time-resolved and quantitative binding studies with other compounds, such as metabolites, in a (patho)physio­logical environment.

## Figures and Tables

**Figure 1:**
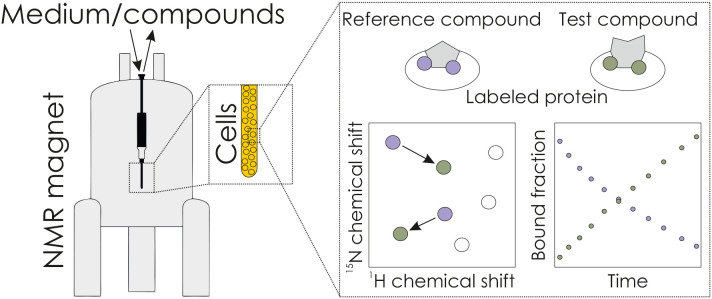
Scheme of in-cell determination of binding affinities using an NMR bioreactor setup.
